# Supplementary material for: Serum proteomic profiling during the periovulatory period identifies preliminary candidate biomarkers of oocyte maturation in deslorelin-induced ovulation in dogs
Source: PeerJ. 2025 Oct 15;13:e20106. doi: 10.7717/peerj.20106 (PMC12535230; doi:10.7717/peerj.20106)
Supplement: Supplemental Information 1 [file peerj-13-20106-s001.docx]

**List of candidate proteins and Gene Ontology terms determined using UniProtKB/Swiss-Prot.**

| Protein names | Gene Names | Gene Ontology (biological process) | Gene Ontology (cellular component) | Gene Ontology (molecular function) | f.value | p.value | -log10 (p) | FDR | Tukey's HSD |
| --- | --- | --- | --- | --- | --- | --- | --- | --- | --- |
| Tubulin-specific chaperone D | TBCD | post-chaperonin tubulin folding pathway, tubulin complex assembly |  | beta-tubulin binding, GTPase activator activity | 34.413 | 8.92E-08 | 7.0495 | 0.000834 | ovulated immature-before ovulation; ovulated mature-before ovulation |
| Coiled-coil domain-containing protein 93 | CCDC93 | endocytic recycling, Golgi to plasma membrane transport | intracellular membrane-bounded organelle | N/A | 27.767 | 5.70E-07 | 6.2441 | 0.002665 | ovulated immature-before ovulation; ovulated mature-ovulated immature |
| WDFY family member 4 | WDFY4 | N/A | N/A | N/A | 18.486 | 1.38E-05 | 4.8591 | 0.04311 | ovulated immature-before ovulation; ovulated mature-ovulated immature |
| Calcium and integrin-binding protein 1 (Calmyrin) | CIB1 | Angiogenesis, cell adhesion, cell differentiation, cell division, spermatogenesis | apical plasma membrane, centrosome, cytoplasm, filopodium tip, growth cone, lamellipodium, nucleus, perikaryon, ruffle membrane, sarcolemma | calcium ion binding | 16.137 | 3.62E-05 | 4.4412 | 0.049164 | ovulated immature-before ovulation; ovulated mature-before ovulation |
| IQ motif containing E | IQCE | N/A | N/A | N/A | 15.655 | 4.45E-05 | 4.3512 | 0.049164 | ovulated mature-before ovulation; ovulated mature-ovulated immature |
| Large ribosomal subunit protein uL23 N-terminal domain-containing protein | RPL23A | translation | ribosomal subunit | rRNA binding, structural constituent of ribosome | 15.63 | 4.50E-05 | 4.3464 | 0.049164 | ovulated immature-before ovulation; ovulated mature-ovulated immature |
| Neuraminidase 4 | NEU4 | glycolipid biosynthetic process | membrane [GO:0016020] | galactosylceramide sulfotransferase activity | 15.585 | 4.59E-05 | 4.3378 | 0.049164 | ovulated mature-before ovulation; ovulated mature-ovulated immature |
| G protein-coupled receptor kinase | GRK3 | Phosphorylation, signal transduction | N/A | ATP binding, G protein-coupled receptor kinase activity | 15.287 | 5.23E-05 | 4.2813 | 0.049164 | ovulated mature-before ovulation; ovulated mature-ovulated immature |
| NFKB inhibitor delta | NFKBID | N/A | N/A | N/A | 15.203 | 5.43E-05 | 4.2652 | 0.049164 | ovulated mature-before ovulation; ovulated mature-ovulated immature |
| Leucine rich repeat containing 4B | LRRC4B | positive regulation of synapse assembly | cerebellar mossy fiber, synaptic membrane | signaling receptor binding | 15.143 | 5.58E-05 | 4.2537 | 0.049164 | ovulated immature-before ovulation; ovulated mature-before ovulation |
| Rho family-interacting cell polarization regulator 2 | RIPOR2 | cell adhesion, cell differentiation, chemotaxis, muscle organ development, negative regulation of signal transduction, sensory perception of sound | apical plasma membrane, cytoplasm, cytoskeleton, filopodium, stereocilium membrane | N/A | 15.06 | 5.78E-05 | 4.2378 | 0.049164 | ovulated mature-before ovulation; ovulated mature-ovulated immature |
